# Comparison of Diaphragmatic Breathing Exercise, Volume and Flow Incentive Spirometry, on Diaphragm Excursion and Pulmonary Function in Patients Undergoing Laparoscopic Surgery: A Randomized Controlled Trial

**DOI:** 10.1155/2016/1967532

**Published:** 2016-07-21

**Authors:** Gopala Krishna Alaparthi, Alfred Joseph Augustine, R. Anand, Ajith Mahale

**Affiliations:** ^1^Department of Physiotherapy, Kasturba Medical College, Manipal University, Bejai, Mangalore 575004, India; ^2^Department of Surgery, Kasturba Medical College, Manipal University, Mangalore 575004, India; ^3^Department of Pulmonary Medicine, Kasturba Medical College, Manipal University, Mangalore 575004, India; ^4^Department of Radiodiagnosis, Kasturba Medical College, Manipal University, Mangalore 575004, India

## Abstract

*Objective.* To evaluate the effects of diaphragmatic breathing exercises and flow and volume-oriented incentive spirometry on pulmonary function and diaphragm excursion in patients undergoing laparoscopic abdominal surgery.* Methodology.* We selected 260 patients posted for laparoscopic abdominal surgery and they were block randomization as follows: 65 patients performed diaphragmatic breathing exercises, 65 patients performed flow incentive spirometry, 65 patients performed volume incentive spirometry, and 65 patients participated as a control group. All of them underwent evaluation of pulmonary function with measurement of Forced Vital Capacity (FVC), Forced Expiratory Volume in the first second (FEV_1_), Peak Expiratory Flow Rate (PEFR), and diaphragm excursion measurement by ultrasonography before the operation and on the first and second postoperative days. With the level of significance set at *p* < 0.05.* Results.* Pulmonary function and diaphragm excursion showed a significant decrease on the first postoperative day in all four groups (*p* < 0.001) but was evident more in the control group than in the experimental groups. On the second postoperative day pulmonary function (Forced Vital Capacity) and diaphragm excursion were found to be better preserved in volume incentive spirometry and diaphragmatic breathing exercise group than in the flow incentive spirometry group and the control group. Pulmonary function (Forced Vital Capacity) and diaphragm excursion showed statistically significant differences between volume incentive spirometry and diaphragmatic breathing exercise group (*p* < 0.05) as compared to that flow incentive spirometry group and the control group.* Conclusion*. Volume incentive spirometry and diaphragmatic breathing exercise can be recommended as an intervention for all patients pre- and postoperatively, over flow-oriented incentive spirometry for the generation and sustenance of pulmonary function and diaphragm excursion in the management of laparoscopic abdominal surgery.

## 1. Introduction

Chest physiotherapy is a common practice in patients undergoing cardiothoracic and abdominal surgery [1]. Abdominal surgery that was previously performed via a large incision is now more commonly performed laparoscopically [2]. The laparoscopic surgeries involve structures such as the gall bladder, colon, small intestine, stomach, liver, and pancreas [1].

In laparoscopy, intraoperative pulmonary changes are due to decreased pulmonary compliance secondary to upward movement of the diaphragm during insufflation and to changes in carbon dioxide (CO_2_) homeostasis secondary to absorption of insufflated CO_2_ from peritoneum [3]. General anesthesia and surgery related pain may lead to changes in the ventilation pattern resulting in the patient taking shallow breaths which reduce the ability to clear sputum from the chest [4–6].

Studies have reported altered pulmonary function after both conventional and laparoscopic abdominal surgeries [7–12]. Postoperative pulmonary dysfunction in laparoscopic surgery is approximately 20% to 25% depending upon the type of surgery [7–9]. Pulmonary dysfunction leads to pulmonary complications which includes atelectasis, pneumonia, tracheobronchial infection, and respiratory failure. These may have an adverse effect on the length of hospital stay [4].

Reduction of pulmonary function, Forced Vital Capacity (FVC), and Forced Expiratory Vital Capacity (FEV_1_) have been reported on the basis of functional alterations [13]. Pathogenesis of postoperative pulmonary dysfunction has been attributed to diaphragmatic function impairment [14].

Chest physiotherapy has been employed as an alternative intervention to reduce occurrence of pulmonary function loss and its complications. Postoperative chest physiotherapy started being implemented in the beginning of the 20th century. It includes breathing exercises, percussion, vibration, splinted huffing/coughing, positioning, and mobilization [15].

Diaphragmatic breathing exercises are used in order to augment diaphragmatic descent while inhalation and diaphragmatic ascent while expiration. The beneficial effects of diaphragmatic breathing are as follows: inflation of the alveoli, reversing postoperative hypoxemia, improvement of ventilation and oxygenation, decreasing the work of breathing, and increasing the degree of excursion of the diaphragm [16, 17].

Mechanical breathing device such as the incentive spirometry (IS) has been introduced into clinical practice [13]. Incentive spirometry encourages the patient to take long, slow deep breath mimicking natural sighing and also provides a visual positive feedback. Incentive spirometers are available either by volume of inspiration (volume-oriented) or flow rate (flow-oriented) [4–6, 18–20].

The flow-oriented incentive spirometer (Triflow device) consists of three chambers in series, each of which contains a ball. When the patient's effort generates a subatmosphere pressure above the ball, it rises in the chamber. An inspiratory flow of 600 mL/s is required to raise the first ball, an inspiratory flow of 900 mL/s is required to elevate the first and second balls, and a flow of 1200 mL/s is required to elevate all three balls. The volume-oriented incentive spirometer is a compact device of 4000 mL capacity and has a one-way valve to prevent exhalation into the unit. A sliding pointer indicates the prescribed inspiratory volume and an inspiratory flow guide coaches the subject to inhale slowly [18–20].

Studies suggest a physiologically significant difference in the effect of the flow- and volume-oriented incentive spirometer. Flow-oriented devices (Triflow device) enforce more work of breathing and increase muscular activity of the upper chest. Volume-oriented devices (Coach 2 device) enforce less work of breathing and improve diaphragmatic activity [6, 18–21].

Earlier studies show that the volumetric incentive spirometer is better in case of cardiac and thoracic surgeries because it provides the appropriate feedback for a slow sustained inspiration and volume [18]. Studies show that slow sustained inspirations are much more effective to promote lung expansion rather than fast inspirations [18]. Studies also show that diaphragmatic breathing exercise encourages more diaphragmatic movement [17, 18].

Gastaldi et al. studied thirty-six subjects, in order to assess the effect of respiratory kinesiotherapy on respiratory muscle strength and pulmonary function following laparoscopic cholecystectomy. Subjects were randomly sorted into two groups: the exercise and the control. Three breathing exercises were performed by seventeen subjects while other nineteen served as a control group. All the subjects were assessed for Maximal Inspiratory Pressure (MIP) and Maximal Expiratory Pressure (MEP), PEF, and spirometry (FVC, FEV_1_, and FEV_1_/FVC). Both groups registered a decrease in all variables on the first day after surgery. On the second postoperative day, the exercise group showed decreased values for all variables. The values then normalized. However, values of all variables for the control group begin to normalize only on the fifth postoperative day [22].

El-Marakby et al. carried out a study on two experimental groups of patients in order to evaluate the effects of aerobic exercise training and incentive spirometry in controlling pulmonary complications following laparoscopic cholecystectomy. One group was given aerobic walking raining and incentive spirometry as well as traditional physical therapy (Group A); the other (Group B) was given traditional physical therapy. Results indicated a significant reduction in heart rate, SaO_2_, and inspiratory capacity for both groups. The researchers concluded that aerobic exercise and incentive spirometry were beneficial in reducing the postoperative pulmonary complications after laparoscopic cholecystectomy [23].

Kundra et al. carried out a comparative study on the effect of preoperative and postoperative incentive spirometry on the pulmonary function of fifty patients who had undergone laparoscopic cholecystectomy. The study group had to carry out incentive spirometry fifteen times before surgery, every four hours, for one week. However, the control group underwent incentive spirometry only during the postoperative period. Pulmonary function was recorded before surgery and 6, 24, and 48 hours postoperatively and at the time of discharge. Result showed that pulmonary function improvement was seen after preoperative incentive Spirometry. The authors concluded that pulmonary function is well-preserved with preoperative than postoperative incentive spirometry [24].

Fagevik Olsén et al. reviewed forty-four studies in order to evaluate the effects of chest physiotherapy interventions in laparoscopic and open abdominal surgery. But the results showed that breathing exercises were efficacious in preventing postoperative pulmonary complications in patients undergoing open surgery. The review also showed that laparoscopic procedures impair respiratory function to a considerably lower degree than open surgery. One study in the review showed that routine treatment is not called for in upper gastrointestinal features such as, for instance, fundoplication and vertical banded gastroplasty [1].

Cattano et al. studied forty-one morbidly obese to assess use of incentive spirometry preoperatively which could help patients to preserve their pulmonary function (inspiratory capacity) better in the postoperative period following laparoscopic bariatric surgery. Subjects were randomly sorted into two groups (the exercise and the control group). The exercise group used the incentive spirometer for ten breaths, five times per day. The control group used incentive spirometer three breaths, once per day. Pulmonary function (inspiratory capacity) was recorded at the day of surgery and postoperative day 1. The author concluded that preoperative use of the incentive spirometer does not lead to significant improvement of pulmonary function (inspiratory capacity) [25].

Various chest physiotherapy techniques are used clinically as part of the routine prophylactic and therapeutic regimen in postoperative respiratory care. However, the efficacy of flow and volume incentive spirometry and diaphragmatic breathing exercise is still controversial [6, 17].

There are no retrievable studies that have been done on the clinical efficacy of diaphragmatic breathing exercise and flow and volume incentive spirometry after laparoscopic abdominal surgery. With this background the present study aim is to compare the effect of diaphragmatic breathing exercise, flow and volume incentive spirometry, on pulmonary function and diaphragm excursion, following laparoscopic surgery.

## 2. Material and Method 

### 2.1. Inclusion Criteria

Inclusion criteria involved subjects of either gender in the age group of 18 to 80 years who were posted for laparoscopic abdominal surgery.

### 2.2. Exclusion Criteria

Exclusion criteria were as follows:

- Patients who had undergone open abdominal surgery and laparoscopic obstetrics and gynecological surgery.
- Patients with unstable hemodynamic parameters (arterial pressure <100 mmHg systolic and <60 mmHg for diastolic and mean arterial pressure (MAP) <80 mmHg).
- Patients with postoperative complications requiring mechanical ventilation.
- Uncooperative patients or patients unable to understand or to use the device properly.
- Patients with inadequate inspiration characterized by vital capacity <10 mL/kg.

### 2.3. Equipment Used

Equipment used was as follows:

- Ultrasonography machine (Voluson730).
- Pulmonary function test machine (EasyOne Plus Portable Diagnostic Spirometer Machine, ndd Medical Technologies, Inc. Massachusetts, USA).
- Flow-oriented incentive spirometry machine (Triflow device, IGNA Medical Devices, Mumbai).
- Volume-oriented incentive spirometry machine (Coach 2 device, Smiths Medical International Ltd., USA).

### 2.4. Procedure

The study was carried out in Kasturba Medical College Hospitals Mangalore over a period of four years starting from January 2011 to December 2014. The study was approved by the Institutional Ethics Committee of Kasturba Medical College Mangalore. Eligible patients were selected based on the inclusion and exclusion criteria. The purpose of study was made clear to each patient and a written informed consent was obtained prior to involving them in the study.

The patients were divided into four groups:

 Flow-oriented incentive spirometry group (Triflow device).
 Volume-oriented incentive spirometry group (Coach 2 device).
 Diaphragmatic breathing exercise group.
 Control group.

The patients were allocated to groups by block randomization done by primary investigator. The entire sample was divided into 13 blocks with 20 patients in each, 5 belonging to each group. Group information was concealed in a sealed opaque envelope and revealed to the patients only after they were recruited into the treatment group or the control group done by primary investigator.

Following the allocation to groups, the patients in the treatment group were visited one day prior to the surgery; preoperative information was offered and, based upon his/her group, flow-oriented incentive spirometry, volume-oriented incentive spirometry, or diaphragmatic breathing exercise was taught to each patient. Other therapies like airway clearance techniques, thoracic expansion exercise, and mobilization were also taught to every patient in all treatment groups (see Steps 1–5). Patients in the control group were not given any treatment or taught any exercises. The treatment protocol for postoperative laparoscopic abdominal surgery is as follows.

**Step 1 .**

The first step is diaphragmatic breathing exercise, flow or volume incentive spirometry (3 sets, 5 repetitions of deep breaths).

**Step 2 .**

The second step is airway clearance techniques (huffing or coughing).

**Step 3 .**

The third step is circulation (foot and ankle pumping, hip and knee bending 10 times each hour).

**Step 4 .**

The fourth step is thoracic expansion exercise (position patient in long sitting in bed/high sitting over the side of the bed).

**Step 5 .**

The fifth step is mobilization:

- Sitting out of the bed in a chair (one hour twice daily).
- Walking (three times per day).
- Stair climbing done before the patient was discharged from the hospital.

An experienced radiologist carried out ultrasonography for diaphragm excursion on the preoperative as well as the 1st and 2nd postoperative day, for all groups.

**Figure 1:**
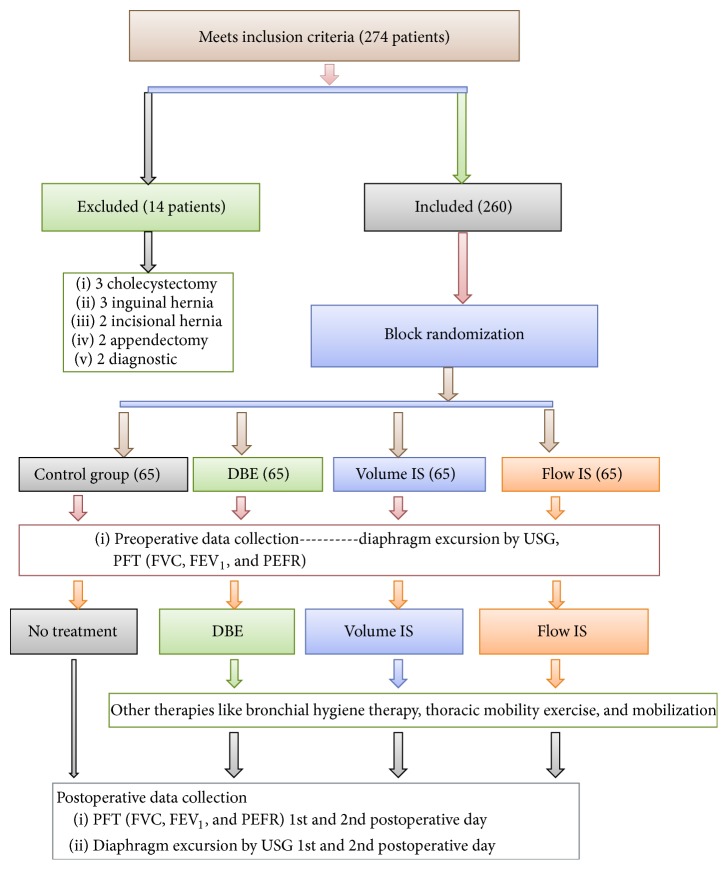
Consort flow diagram of the study.

Pulmonary function tests (PFT) measured the following variables: Forced Vital Capacity (FVC), Forced Expiratory Volume in the first second (FEV_1_), Peak Expiratory Flow Rate (PEFR). These were taken on the preoperative day and 1st and the 2nd postoperative day, for all groups. These measurements were taken by the primary investigator (Figure 1, flowchart).

## 3. Description of Outcome Measures

### 3.1. Diaphragm Excursion

The patient lays in the supine position and diaphragm movements were recorded in the B-Mode. The probe was positioned between the midclavicular and anterior axillary lines, in the subcostal area, so that the ultrasound beam entered the posterior third of the right hemidiaphragm perpendicularly. The procedure began at the end of normal expiration with the subjects being instructed to inhale as deeply as possible. A fixed point at the edge of the image on the screen and the diaphragm margin at maximal inspiration and again at maximal expiration served as reference points between which measurements were made, with the average of three values being taken for both maximal inspiration and maximal expiration [26, 27].

### 3.2. Pulmonary Function Test

Pulmonary function test procedures (EasyOne Plus Portable Diagnostic Spirometer Machine) were carried out according to the American Thoracic Society/European Respiratory Society guidelines [28]. The following variables have been recorded: Forced Vital Capacity (FVC), Forced Expiratory Volume in the first second (FEV_1_), and Peak Expiratory Flow Rate (PEFR) the best value of 3 acceptable tests [29].

### 3.3. Treatment Procedures

#### 3.3.1. Methods to Perform Flow-Oriented and Volume-Oriented Incentive Spirometry

The patient was placed in a semirecumbent position (45°), with a pillow under the knees. The patient was instructed to inhale with a slow and deep sustained breath, holding it for a minimum of 5 seconds and then to exhale passively in order to avoid any forceful expiration. First, the patient was given demonstration and then asked to perform in order to ensure that she/he understood the process [15, 17]. The patient was instructed to hold the spirometer upright and to perform flow-oriented incentive spirometry by inhaling slowly and thereby raising the ball, followed by volume incentive spirometry in order to raise the piston or plate in the chamber to the set target [19, 20].

The patient was instructed to perform 3 sets of 5 repeated deep breaths. This had to be performed by the patient every waking hour. The therapist administered the exercise four times a day and the patient was instructed to perform the same for the rest of the day [19]. The patient was asked to keep a record of the exercise performed by entering in a log book which was provided beforehand.

#### 3.3.2. Method to Perform Diaphragmatic Breathing Exercise

The patient assumed a semi-Fowler's position (back and head are fully supported and abdominal wall is relaxed) and performed diaphragmatic breathing. The therapist placed his hands just below the anterior costal margin, on the rectus abdominis, while the patient was instructed to inhale slowly and deeply through the nose, from functional residual capacity to total lung capacity with a three-second inspiratory hold. The patient was then instructed to relax the shoulders, keep the upper chest quiet in order that the abdomen be raised a little. The Patient was then instructed to exhale slowly through the mouth [16, 28].

The Patient was made to experience a slight rise and subsequent fall of the abdomen during inspiration and expiration, by placing his or her own hand below the anterior costal margin. The Patient was instructed to perform 3 sets of 5 deep breaths with the therapist administering them four times a day and the patient being instructed to perform the same once every waking hour for the rest of the day. In between the repetitions of the diaphragmatic breathing exercise, the patient was told to breathe normally [16, 28]. The patient was asked to keep a record of the exercise performed by entering in a log book which was provided beforehand.

### 3.4. Data Analysis

#### 3.4.1. Sample Size

The sample size was calculated based on the values obtained from pulmonary function test in a pilot study (20 subjects, 5 in each group) [30, 31]. The following formula was used for calculating the same:

(1) $$\begin{array}{c} n=2{\left(\;\frac{Z\alpha +Z\beta }{D/S}\;\right)}^{2}, \end{array}$$

where *n* is the number of subjects in each group and *Zα* and *Zβ* are constants and they are substituted. Selected power for the study was 90% and *D* is effect size which is the absolute value of the difference in means and represents what is considered a clinically meaningful or practically important difference in means.

*D* is taken from the pilot study which used the same variable, which compared pulmonary function test in subjects, and *S* is the standard deviation of the means. The sample size is 65 in each group (total 260 subjects).

Data was analyzed using SPSS package version 21. ANOVA and post hoc analysis (Bonferroni's *t*-test) were carried out to verify the within-groups differences. Between-groups differences were compared using two-factor ANOVA.

## 4. Results 

We selected 274 patients posted for laparoscopic abdominal surgery, of which 260 were included in the study. Fourteen patients were excluded because they were converted to an open surgical procedure. There were 195 patients in the intervention groups and 65 in the control group.

Baseline demographic characteristics of the participants such as age, height, weight, BMI, risk factors, and duration of surgery are presented in Table 1. There were no statistically significant differences between the groups. Data about Patients who underwent different types of laparoscopic abdominal surgery are summarized in Table 1. Of the 260 patients included, 140 patients underwent cholecystectomy, 53 hernioplasty, 43 appendectomy, 11 umbilical hernia repair, 8 laparoscopic diagnostic, 3 bariatric surgery, and 2 hemicolectomy.

Forced Vital Capacity (FVC) was compared within the intervention groups and the control group before and after operation, and the same is summarized in Table 2. There was a statistically significant decrease in Forced Vital Capacity (FVC) in the 1st and 2nd post-op day when compared with the preoperative period in all groups.

Forced Expiratory Volume in one second (FEV_1_) was compared within the intervention groups and the control group before and after the operation and is summarized in Table 3. There was a statistically significant decrease in Forced Expiratory Volume at the end of the first second (FEV_1_) on the 1st and 2nd postoperative day when compared with the preoperative period in all groups.

Peak Expiratory Flow Rates (PEFR) were compared with the intervention groups and control group before and after operation and are summarized in Table 4. In all groups there was a statistically significant decrease in Peak Expiratory Flow Rate (PEFR) on the 1st and 2nd postoperative day compared to the preoperative period.

Diaphragm excursions were compared within intervention groups and the control group before and after operation and are summarized in Table 5. There was a statistically significant decrease in diaphragm excursion in the 1st and 2nd postoperative period when compared with the preoperative period in all groups except in diaphragmatic breathing exercise group and volume incentive spirometry group which almost came back to normal.

Forced Vital Capacity (FVC), Forced Expiratory Volume in one second (FEV_1_), Peak Expiratory Flow Rate, and diaphragm excursion were compared between the intervention groups and the control group during the preoperative and 2nd postoperative day and are summarized in Table 6.

There was a statistically significant difference between intervention groups (diaphragmatic breathing exercise group and volume incentive spirometry group) and control group in terms of Forced Vital Capacity (FVC) and diaphragm excursion (*p* < 0.001), the said variables being significantly lower in the control group than in the diaphragmatic breathing exercise group and volume incentive spirometry group.

## 5. Discussion 

The main purpose of this study was to compare diaphragmatic breathing exercise, flow- and volume incentive spirometry, on pulmonary function and diaphragmatic excursion in patients undergoing laparoscopic abdominal surgery. To the best of our knowledge, this study is the first to compare the effects of diaphragmatic breathing exercise with two different kinds of incentive spirometry and also against a control group. There were 65 patients included in each group and the four groups were homogenous in terms of all demographic parameters. In our study we found that diaphragmatic breathing exercise and volume incentive spirometry improve lung function and diaphragm excursion in patients undergoing laparoscopic abdominal surgery.

In our study pulmonary function (FVC, FEV_1_, and PEFR) and diaphragm excursion showed a decrease on the 1st postoperative day when compared to the preoperative values in all four groups with an average decrease of 27% in Forced Vital Capacity, 28% in Forced Expiratory Volume in one second, 37% in Peak Expiratory Flow Rate, and 28% in diaphragm excursion. The present study finding of reduction in pulmonary function during postoperative day is similar to those reported in a previous study [3, 21–24].

Our results are in accordance with Schauer et al. who found 30% to 38% reduction in postoperative pulmonary function (FVC, FEV_1_, and FEF25%–75%) in laparoscopic cholecystectomy [9]. Karayiannakis et al. found 22% of FVC and 19% of FEV_1_ reduction after laparoscopic cholecystectomy [32]. Ramos et al. found 20% to 30% reduction in postoperative pulmonary function (FVC and FEV_1_) in laparoscopic cholecystectomy [33]. Ravimohan et al. found 21% to 31% reduction in postoperative day pulmonary function variables (FVC, FEV_1_, and FEF25%–75%) in laparoscopic cholecystectomy [7].

Possible reasons for decrease in pulmonary function and diaphragm excursion during the postoperative period in laparoscopic abdominal surgery are as follows. During the postoperative period, patients exhibit shallow breathing without the intermittent sigh or breaths which are inspired approximately ten times an hour. Patients will breathe shallowly which leads to a decrease in ventilation to dependent lung regions [7, 32, 33]. In the present study, reduced pulmonary function (FVC, FEV_1_, and PEFR) and diaphragm excursion in postoperative laparoscopic abdominal surgery subjects might be due to postoperative pain, location of surgical ports, along with anaesthetic, analgesic usage [7, 34].

The effects of general anaesthesia on distribution of ventilation and chest wall and lung mechanics lead to ventilation-perfusion mismatch, increased dead space, shunt, and hypoxemia [9, 35, 36]. Narcotic/opioid analgesics and other drugs affect the central regulation of breathing, changing the neural drive of the upper airway and chest wall muscles, which lead to hypoventilation, a diminished sensitivity of the respiratory center to carbon dioxide stimulation, an increase of obstructive breathlessness, the suppression of the cough reflex, and irregular mucus production [37].

The location of surgical ports involves trauma near the diaphragm and chest wall/ribs, leading to postoperative incisional pain and reflex inhibition of the phrenic nerve and diaphragmatic reflex paresis resulting in functional disruption of respiratory muscle movement. In addition, when patients remain lying down for long periods during the postoperative period their abdominal content limits diaphragmatic movement [34].

Several studies found that diaphragmatic dysfunction is due to gas insufflation in the abdominal cavity which might also be responsible for the increase of resistance and reduced diaphragmatic excursion, leading to reduced lung volume [38]. All these factors lead to a change in postoperative lung function usually resulting in development of a restrictive pattern and decreased diaphragm excursion in laparoscopic abdominal surgery.

Our results are in accordance with Ford et al., who showed that reduction in inspiratory muscle activity, mainly the diaphragm, was the main determinant of impaired pulmonary function. Diaphragm dysfunction may be due to reflex inhibition of efferent phrenic activity [39]. Several studies suggested that laparoscopic abdominal surgery causes reflex inhibition of the phrenic nerve which might lead to shallow breaths and reduced pulmonary ventilation [34]. Erice et al. explained reduced pulmonary ventilation mainly due to decreased inspiratory muscle activity [40]. Lunardi et al. showed a decrease of 27% in the respiratory muscular activity of patients who underwent laparoscopy abdominal surgery [41].

Possible reasons for improved pulmonary function and diaphragm excursion in the diaphragmatic breathing exercise group are as follows. The present study showed that the diaphragmatic breathing exercise group was able to improve pulmonary mechanics thus leading to a beneficial effect on pulmonary function (FVC) and diaphragm excursion. Diaphragmatic breathing exercise improves diaphragmatic descent and diaphragmatic ascent during inspiration and expiration, respectively. Slower deep inspiration ensures more even distribution of air throughout the lung, particularly to the dependent lung [16]. The physiological effects of diaphragmatic breathing exercise are that breathing through full vital capacity and holding for 3–5 seconds ensure full inflation of the lungs thus opening up alveoli which have low volume and stimulating the production of surfactant. Diaphragmatic breathing exercise will also decrease activity of accessory muscles, ensure that breathing patterns are as close to normal as possible, and also reduce the work of breathing [16, 31].

Our results are in accordance with the findings of Tahir et al. who showed that diaphragmatic breathing exercise will improve basal ventilation [42]. Weber and Prayar and Menkes and Britt found that diaphragmatic breathing exercise will improve tidal volume and also facilitate secretion removal [43, 44]. Blaney and Sawyer observed that tactile stimulation over the subject's lower costal margin as well as verbal instruction served to significantly increase diaphragmatic movement during diaphragmatic breathing exercises [45]. Manzano et al. found that diaphragmatic breathing exercise was able to improve pulmonary mechanics and lead to beneficial effect on Forced Vital Capacity (FVC) [46]. Grams et al. evaluated the efficacy of diaphragmatic breathing exercise for the prevention of postoperative pulmonary complications and for the recovery of pulmonary mechanics and found that diaphragmatic breathing exercise appeared to be more effective [17].

Possible reasons for improved pulmonary function and diaphragmatic excursion in the volume incentive spirometry group are as follows. The present study showed that the volume incentive spirometry group also had improved pulmonary mechanics that led to a beneficial effect on pulmonary function (FVC) and diaphragm excursion. After laparoscopic abdominal surgery, it may be hard to take a deep breath and if patients do not breathe deeply it may lead to postoperative pulmonary complications. The volume incentive spirometer is a mechanical device used to take slow, deep long breaths that encourage patients to breathe to total lung capacity, to sustain that inflation and open up collapsed alveoli [18].

The volume incentive spirometer will be more “physiological” because the training volume is constant until it reaches the maximum inspiratory capacity (level preset by physiotherapist). It provides a low level of resistance training while minimizing the potential fatigue to the diaphragm [19]. Our study results are in accordance with Paisani et al. who showed that when volume incentive spirometry was performed with low inspiratory flow it promoted diaphragmatic excursion and improved the expansion of the basal area of chest wall [21]. Minschaert et al. observed that patients treated with incentive spirometry would have early recovery of the pulmonary volume [47]. Kundra et al. found that the use of incentive spirometry in the preoperative period leads to greater improvement in the lung functions than if given in the postoperative period. So use of the volume incentive spirometer will result in active recruitment of the diaphragm and other inspiration muscles which may lead to improved pulmonary function and diaphragm excursion [24].

*Limitation of the Study*. There was no blinding in the study procedure; the same investigator who randomized the patients into the experimental groups and the control group measured the outcome variables (pulmonary function test) and the same investigator taught the exercises to all experimental groups. Diaphragm excursion measurement was not done by the same radiologist throughout the study and the finding would have been confounded by the expertise of professional. Type of anaesthesia, analgesia, and postoperative pain was not recorded which could affect the findings. There was no follow-up in the study as all patients were discharged on the 2nd postoperative day. As a result we are unaware which group values returned to normal. Patient adherence to the intervention programs was recorded by providing a log book to each subject, in which they had to make an entry the very time they did the prescribed technique but there is no way to verify the authenticity of these entries.

## 6. Suggestions for the Future Research 

Future research could be directed at long-term follow-up to see which group sustains improvement for a long duration and the functional aspect of recovery. Future studies can be carried out to compare the effect of the techniques on patients who have undergone upper and lower abdominal laparoscopic surgeries, using a larger sample size. Effect of combining therapy like incentive spirometer and diaphragmatic breathing exercise can be studied on laparoscopic abdominal surgery patients. Future research can be done by assessing and using respiratory muscle strength and patient comfort with different technique as an outcome in laparoscopic abdominal surgery. Similar studies can be conducted on patients following open abdominal surgeries and cardiac and thoracic surgeries.

## 7. Clinical Implication 

Based on the results of the study we strongly recommend the following:

 Volume-oriented incentive spirometry and diaphragmatic breathing exercise can be recommended for all patients preoperatively and postoperatively over flow-oriented incentive spirometry as an intervention for the generation and sustenance of pulmonary function and diaphragm excursion in the management of laparoscopic abdominal surgery.

## 8. Conclusion

(i) From our study we conclude that in laparoscopic abdominal surgery patients there is a significant decrease in pulmonary function (FVC, FEV_1_, and PEFR) and diaphragm excursion in all four groups on the 1st postoperative day when compared with the preoperative day.
(ii) A greater improvement in pulmonary function and diaphragm excursion between the first and second postoperative day was seen in all experimental groups when compared to the control group.
(iii) From our study we conclude that pulmonary function and diaphragm excursion was better preserved in the diaphragmatic breathing exercise group and volume incentive spirometry group when compared with the flow incentive spirometry group and the control group.

## Figures and Tables

**Table 1:** Demographic characteristics of subjects undergoing laparoscopic abdominal surgery.

Variables	Intervention groups			Control group (*N* = 65)	*p* value (<0.05)
	Diaphragmatic breathing exercise (*N* = 65)	Flow incentive spirometry (*N* = 65)	Volume incentive spirometry (*N* = 65)		
Age (years) (mean ± SD)	41.8 ± 13.6	49.5 ± 16.1	45.5 ± 15.3	46.2 ± 16.4	0.055 NS
Gender (*n*) M : F	47 : 18	37 : 28	33 : 32	40 : 25	
Height (cm) (mean ± SD)	166.8 ± 11.6	165.0 ± 11.1	163.7 ± 10.0	163.5 ± 9.9	0.268 NS
Weight (kg) (mean ± SD)	65.2 ± 12.5	63.8 ± 12.6	62.7 ± 19.7	60.2 ± 12.3	0.266 NS
BMI (mean ± SD)	23.3 ± 3.3	23.0 ± 4.9	23.4 ± 6.1	22.5 ± 3.5	0.660 NS
H/o of smoking	3	2	3	1	
H/o of cardiac disease	1	2	2	2	
H/o of hypertension	6	9	6	3	
H/o of diabetes	2	3	5	7	
H/o of asthma	1	Nil	1	3	
Duration of surgery (Hrs)	1.78 ± 0.67	1.89 ± 0.59	1.76 ± 0.66	1.80 ± 0.53	0.63 NS

Type of laparoscopic abdominal surgery					Total number

Cholecystectomy	28	39	44	29	140
Hernioplasty	14	15	11	13	53
Umbilical hernia repair	5	1	2	3	11
Appendectomy	16	6	6	15	43
Laparoscopic diagnostic	2	1	Nil	5	8
Bariatric surgery	Nil	1	2	Nil	3
Hemicolectomy	Nil	2	Nil	Nil	2

**Table 2:** Comparison of Forced Vital Capacity (FVC) before and after the laparoscopic abdominal surgery in the intervention groups and control group.

Forced Vital Capacity (FVC) (liters (L))	Preoperative (mean ± SD)	Postoperative 1st day (mean ± SD)	Postoperative 2nd day (mean ± SD)	Preoperative versus postoperative 1st day (mean difference)	Postoperative 1st day versus postoperative 2nd day (mean difference)	Preoperative versus postoperative 2nd day (mean difference)
Diaphragmatic breathing exercise (*n* = 65)	2.83 ± .79	2.19 ± .84	2.55 ± .79	0.63 (22.4%) *p* < 0.001^*∗∗*^	−0.35 (−16.2%) *p* < 0.001^*∗∗*^	0.28 (9.8%) *p* < 0.001^*∗∗*^

Flow incentive spirometry (*n* = 65)	2.50 ± .76	1.72 ± .70	2.13 ± .71	0.77 (31.0%) *p* < 0.001^*∗∗*^	−0.40 (−23.6%) *p* < 0.001^*∗∗*^	0.37 (14.7%) *p* < 0.001^*∗∗*^

Volume incentive spirometry (*n* = 65)	2.50 ± .73	1.86 ± .64	2.22 ± .70	0.64 (25.6%) *p* < 0.001^*∗∗*^	−0.36 (−19.4%) *p* < 0.001^*∗∗*^	0.28 (11.1%) *p* < 0.001^*∗∗*^

Control group (*n* = 65)	2.51 ± .80	1.78 ± .65	2.02 ± .67	0.73 (29.2%) *p* < 0.001^*∗∗*^	−0.24 (−13.7%) *p* < 0.001^*∗∗*^	0.49 (19.5%) *p* < 0.001^*∗∗*^

% change. ^*∗∗*^Highly significant at *p* < 0.001 level.

**Table 3:** Comparison of Forced Expiratory Volume in one second (FEV_1_) before and after the laparoscopic abdominal surgery in the intervention groups and control group.

Forced Expiratory Volume in one second (FEV_1_) (liters (L))	Preoperative (mean ± SD)	Postoperative 1st day (mean ± SD)	Postoperative 2nd day (mean ± SD)	Preoperative versus postoperative 1st day (mean difference)	Postoperative 1st day versus postoperative 2nd day (mean difference)	Preoperative versus postoperative 2nd day (mean difference)
Diaphragmatic breathing exercise (*n* = 65)	2.34 ± .70	1.76 ± .72	2.02 ± .69	0.57 (24.5%) *p* < 0.001^*∗∗*^	−0.25 (−14.3%) *p* < 0.001^*∗∗*^	0.32 (13.7%) *p* < 0.001^*∗∗*^

Flow incentive spirometry (*n* = 65)	2.06 ± .68	1.42 ± .64	1.74 ± .64	0.63 (30.9%) *p* < 0.001^*∗∗*^	−0.32 (−22.5%) *p* < 0.001^*∗∗*^	0.31 (15.3%) *p* < 0.001^*∗∗*^

Volume incentive spirometry (*n* = 65)	2.08 ± .64	1.53 ± .55	1.82 ± .64	0.55 (26.3%) *p* < 0.001^*∗∗*^	−0.29 (−19.1%) *p* < 0.001^*∗∗*^	0.25 (12.2%) *p* < 0.001^*∗∗*^

Control group (*n* = 65)	2.06 ± .67	1.42 ± .55	1.62 ± .59	0.64 (31.0%) *p* < 0.001^*∗∗*^	−0.20 (−14.3%) *p* < 0.001^*∗∗*^	0.43 (21.1%) *p* < 0.001^*∗∗*^

% change. ^*∗∗*^Highly significant at *p* < 0.001 level.

**Table 4:** Comparison of Peak Expiratory Flow Rate (PEFR) before and after the laparoscopic abdominal surgery in the intervention groups and the control group.

Peak Expiratory Flow Rate (PEFR) L/s	Preoperative (mean ± SD)	Postoperative 1st day (mean ± SD)	Postoperative 2nd day (mean ± SD)	Preoperative versus postoperative 1st day (mean difference)	Postoperative 1st day versus postoperative 2nd day (mean difference)	Preoperative versus postoperative 2nd day (mean difference)
Diaphragmatic breathing exercise (*n* = 65)	5.83 ± 2.1	3.74 ± 1.8	4.78 ± 2.0	2.09 (35.8%) *p* < 0.001^*∗∗*^	−1.04 (−27.8%) *p* < 0.001^*∗∗*^	1.04 (17.9%) *p* < 0.001^*∗∗*^

Flow incentive spirometry (*n* = 65)	5.21 ± 2.0	2.95 ± 1.3	4.04 ± 1.5	2.25 (43.2%) *p* < 0.001^*∗∗*^	−1.08 (−36.6%) *p* < 0.001^*∗∗*^	1.17 (22.4%) *p* < 0.001^*∗∗*^

Volume incentive spirometry (*n* = 65)	5.52 ± 1.8	3.52 ± 1.3	4.50 ± 1.7	2.00 (36.1%) *p* < 0.001^*∗∗*^	−0.97 (−27.6%) *p* < 0.001^*∗∗*^	1.02 (18.5%) *p* < 0.001^*∗∗*^

Control group (*n* = 65)	5.15 ± 1.8	3.26 ± 1.3	3.89 ± 1.5	1.88 (36.6%) *p* < 0.001^*∗∗*^	−0.62 (−19.2%) *p* < 0.001^*∗∗*^	1.25 (24.4%) *p* < 0.001^*∗∗*^

% change. ^*∗∗*^Highly significant at *p* < 0.001 level.

**Table 5:** Comparison of diaphragm excursion before and after the laparoscopic abdominal surgery in the intervention groups and the control group.

Diaphragm excursion (cm)	Preoperative (mean ± SD)	Postoperative 1st day (mean ± SD)	Postoperative 2nd day (mean ± SD)	Preoperative versus postoperative 1st day (mean difference)	Postoperative 1st day versus postoperative 2nd day (mean difference)	Preoperative versus postoperative 2nd day (mean difference)
Diaphragmatic breathing exercise (*n* = 65)	4.2 ± .90	3.3 ± .91	4.1 ± .99	0.8 (20.6%) *p* < 0.001^*∗∗*^	−0.7 (−22.2%) *p* < 0.001^*∗∗*^	0.1 (2.9%) *p* > 0.20^#^

Flow incentive spirometry (*n* = 65)	4.0 ± 1.0	2.9 ± 1.0	3.7 ± 1.0	1.1 (27.0%) *p* < 0.001^*∗∗*^	−0.7 (−24.7%) *p* < 0.001^*∗∗*^	0.3 (8.9%) *p* < 0.001^*∗∗*^

Volume incentive spirometry (*n* = 65)	4.0 ± 0.8	3.1 ± 0.8	3.9 ± 0.9	0.9 (23.7%) *p* < 0.001^*∗∗*^	−0.8 (−26.4%) *p* < 0.001^*∗∗*^	0.1 (3.5%) *p* > 0.39^#^

Control group (*n* = 65)	4.0 ± 1.0	2.8 ± 0.8	3.4 ± 0.9	1.1 (28.4%) *p* < 0.001^*∗∗*^	−0.5 (−19.0%) *p* < 0.001^*∗∗*^	0.5 (14.8%) *p* < 0.001^*∗∗*^

% change. ^#^Not significant at *p* > 0.05. ^*∗∗*^Highly significant at *p* < 0.001 level.

**Table 6:** Showing difference between preoperative and postoperative 2nd day between intervention groups and control group of Forced Vital Capacity, Forced Expiratory Volume in one second, Peak Expiratory Flow Rate, and diaphragm excursion.

Preoperative minus postoperative 2nd day (mean difference)	Forced Vital Capacity (FVC) (liters (L))	Forced Expiratory Volume in one second (FEV_1_) (liters (L))	Peak Expiratory Flow Rate (PEFR) L/s	Diaphragm excursion (cm)
Diaphragmatic breathing exercise group versus flow incentive spirometry group	−0.09 *p* value 1.00^#^	0.00 *p* value 1.00^#^	−0.12 *p* value 1.00^#^	−0.23 *p* value 0.16^#^

Diaphragmatic breathing exercise group versus volume incentive spirometry group	−0.00 *p* value 1.00^#^	0.06 *p* value 1.00^#^	0.02 *p* value 1.00^#^	−0.15 *p* value 1.00^#^

Diaphragmatic breathing exercise group versus control group	−0.21 *p* value 0.03^*∗*^	−0.11 *p* value 0.85^#^	−0.21 *p* value 1.00^#^	−0.46 *p* value < 0.001^*∗∗*^

Flow incentive spirometry group versus volume incentive spirometry group	0.88 *p* value 1.00^#^	0.06 *p* value 1.00^#^	0.14 *p* value 1.00^#^	0.22 *p* value 0.23^#^

Flow incentive spirometry group versus control group	−0.12 *p* value 0.66^#^	−0.11 *p* value 0.75^#^	−0.08 *p* value 1.00^#^	−0.23 *p* value 0.20^#^

Volume incentive spirometry group versus control group	−0.21 *p* value 0.03^*∗*^	−0.17 *p* value 0.12^#^	−0.23 *p* value 1.00^#^	−0.45 *p* value < 0.001^*∗∗*^

^#^Not significant at *p* > 0.05. ^*∗*^Significant at *p* < 0.05 level. ^*∗∗*^Highly significant at *p* < 0.001 level.

## References

[1] Fagevik Olsén M., Josefson K., Lönroth H. (1999). Chest physiotherapy does not improve the outcome in laparoscopic fundoplication and vertical-banded gastroplasty. *Surgical Endoscopy*.

[2] Denehy L., Browing L., Partridge C. (2007). Abdominal surgery: the evidence for physiotherapy intervention. *Recent Advances in Physiotherapy*.

[3] Wahba R. W. M., Béïque F., Kleiman S. J. (1995). Cardiopulmonary function and laparoscopic cholecystectomy. *Canadian Journal of Anaesthesia*.

[4] Guimarães M. M., El Dib R., Smith A. F., Matos D. (2009). Incentive spirometry for prevention of postoperative pulmonary complications in upper abdominal surgery. *Cochrane Database of Systematic Reviews*.

[5] Carvalho C. R. F., Paisani D. M., Lunardi A. C. (2011). Incentive spirometry in major surgeries: a systematic review. *Brazilian Journal of Physical Therapy*.

[6] do Nascimento Junior P., Módolo N. S. P., Andrade S., Guimarães M. M. F., Braz L. G., El Dib R. (2014). Incentive spirometry for prevention of postoperative pulmonary complications in upper abdominal surgery. *The Cochrane Database of Systematic Reviews*.

[7] Ravimohan S. M., Kaman L., Jindal R., Singh R., Jindal S. K. (2005). Postoperative pulmonary function in laparoscopic versus open cholecystectomy: a prospective, comparative study. *Indian Journal of Gastroenterology*.

[8] Frazee R. C., Roberts J. W., Okeson G. C. (1991). Open versus laparoscopic cholecystectomy. A comparison of postoperative pulmonary function. *Annals of Surgery*.

[9] Schauer P. R., Luna J., Ghiatas A. A. (1993). Pulmonary function after laparoscopic cholecystectomy. *Surgery*.

[10] Putensen-Himmer G., Putensen C., Lammer H., Lingnau W., Aigner F., Benzer H. (1992). Comparison of postoperative respiratory function after laparoscopy or open laparotomy for cholecystectomy. *Anesthesiology*.

[11] Hasukić S., Mesić D., Dizdarević E., Keser D., Hadziselimović S., Bazardzanović M. (2002). Pulmonary function after laparoscopic and open cholecystectomy. *Surgical Endoscopy*.

[12] Osman Y., Fusun A., Serpil A. (2009). The comparison of pulmonary functions in open versus laparoscopic cholecystectomy. *Journal of the Pakistan Medical Association*.

[13] Simonneau G., Vivien A., Sartene R. (1983). Diaphragm dysfunction induced by upper abdominal surgery. Role of postoperative pain. *American Review of Respiratory Disease*.

[14] Chuter T. A. M., Weissman C., Mathews D. M., Starker P. M. (1990). Diaphragmatic breathing maneuvers and movement of the diaphragm after cholecystectomy. *Chest*.

[15] Pasquina P., Tramèr M. R., Granier J.-M., Walder B. (2006). Respiratory physiotherapy to prevent pulmonary complications after abdominal surgery: a systematic review. *Chest*.

[16] Nancy H., Tecklin J. S., Irwin S., Tecklin J. S. (1995). Respiratory treatment. *Cardiopulmonary Physical Therapy; A Guide to Practice*.

[17] Grams S. T., Ono L. M., Noronha M. A., Schivinski C. I. S., Paulin E. (2012). Breathing exercises in upper abdominal surgery: a systematic review and meta-analysis. *Brazilian Journal of Physical Therapy*.

[18] Agostini P., Singh S. (2009). Incentive spirometry following thoracic surgery: what should we be doing?. *Physiotherapy*.

[19] Restrepo R. D., Wettstein R., Wittnebel L., Tracy M. (2011). AARC Clinical Practice Guidelines. Incentive spirometry: 2011. *Respiratory Care*.

[20] Dean R. H., Richard D. B., Richard D. B., Dean R. H., Robert L. C. (1995). Devices for chest physiotherapy, incentive spirometry and intermittent positive-pressure breathing. *Respiratory Care Equipment*.

[21] Paisani D. D. M., Lunardi A. C., da Silva C. C. B. M., Cano Porras D., Tanaka C., Fernandes Carvalho C. R. (2013). Volume rather than flow incentive spirometry is effective in improving chest wall expansion and abdominal displacement using optoelectronic plethysmography. *Respiratory Care*.

[22] Gastaldi A. C., Magalhães C. M. B., Baraúna M. A., Silva E. M. C., Souza H. C. D. (2008). Benefits of postoperative respiratory kinesiotherapy following laparoscopic cholecystectomy. *Revista Brasileira de Fisioterapia*.

[23] El-Marakby A. A., Darwiesh A., Anwar E., Mostafa A., Jad A. (2013). Aerobic exercise training and incentive spirometry can control postoperative pulmonary complications after laparoscopic cholecystectomy. *Middle East Journal of Scientific Research*.

[24] Kundra P., Vitheeswaran M., Nagappa M., Sistla S. (2010). Effect of preoperative and postoperative incentive spirometry on lung functions after laparoscopic cholecystectomy. *Surgical Laparoscopy, Endoscopy and Percutaneous Techniques*.

[25] Cattano D., Altamirano A., Vannucci A., Melnikov V., Cone C., Hagberg C. A. (2010). Preoperative use of incentive spirometry does not affect postoperative lung function in bariatric surgery. *Translational Research*.

[26] Ayoub J., Cohendy R., Prioux J. (2001). Diaphragm movement before and after cholecystectomy: a sonographic study. *Anesthesia and Analgesia*.

[27] Boussuges A., Gole Y., Blanc P. (2009). Diaphragmatic motion studied by M-mode ultrasonography: methods, reproducibility, and normal values. *Chest*.

[28] Silva Y. R., Li S. K., Rickard M. J. F. X. (2013). Does the addition of deep breathing exercises to physiotherapy-directed early mobilisation alter patient outcomes following high-risk open upper abdominal surgery? Cluster randomised controlled trial. *Physiotherapy (United Kingdom)*.

[29] Miller M. R., Hankinson J., Brusasco V. (2005). Standardisation of spirometry. *European Respiratory Journal*.

[30] Alaparthi G. K., Augustine A. J., Anand R., Mahale A. (2013). Chest physiotherapy during immediate postoperative period among patients undergoing laparoscopic surgery-a Randomized Controlled Pilot Trail. *International Journal of Biomedical and Advance Research*.

[31] Alaparthi G. K., Augustine A. J., Anand R., Mahale A. (2013). Comparison of flow and volume oriented incentive spirometry on lung function and diaphragm movement after laparoscopic abdominal surgery: a randomized clinical pilot trial. *International Journal of Physiotherapy*.

[32] Karayiannakis A. J., Makri G. G., Mantzioka A., Karousos D., Karatzas G. (1996). Postoperative pulmonary function after laparoscopic and open cholecystectomy. *British Journal of Anaesthesia*.

[33] Ramos G. C., Pereira E., Gabriel Neto S., de Oliveira E. C. (2009). Pulmonary function after laparoscopic cholecystectomy and abbreviated anesthetic-surgical time. *Revista do Colegio Brasileiro de Cirurgioes*.

[34] Bhat S., Katoch A., Kalsotra L., Chrungoo R. K. (2007). A prospective comparative trial of post-operative pulmonary function: laparascopic versus open cholecystectomy. *JK Science*.

[35] Ali J., Weisel R. D., Layug A. B., Kripke B. J., Hechtman H. B. (1974). Consequences of postoperative alterations in respiratory mechanics. *The American Journal of Surgery*.

[36] Wahba R. W. M. (1991). Perioperative functional residual capacity. *Canadian Journal of Anaesthesia*.

[37] Gamsu G., Singer M. M., Vincent H. H., Berry S., Nadel J. A. (1976). Postoperative impairment of mucous transport in the lung. *American Review of Respiratory Disease*.

[38] Joris J., Kaba A., Lamy M. (1997). Postoperative spirometry after laparoscopy for lower abdominal or upper abdominal surgical procedures. *British Journal of Anaesthesia*.

[39] Ford G. T., Whitelaw W. A., Rosenal T. W., Cruse P. J., Guenter C. A. (1983). Diaphragm function after upper abdominal surgery in humans. *American Review of Respiratory Disease*.

[40] Erice F., Fox G. S., Salib Y. M., Romano E., Meakins J. L., Magder S. A. (1993). Diaphragmatic function before and after laparoscopic cholecystectomy. *Anesthesiology*.

[41] Lunardi A. C., Paisani D. D. M., Tanaka C., Carvalho C. R. F. (2013). Impact of laparoscopic surgery on thoracoabdominal mechanics and inspiratory muscular activity. *Respiratory Physiology & Neurobiology*.

[42] Tahir A. H., George R. B., Weill H., Adriani J. (1973). Effects of abdominal surgery upon diaphragmatic function and regional ventilation. *International Surgery*.

[43] Weber B. A., Prayar J., Webber B. A., Prayar J. (1993). Physiotherapy skills: techniques and adjuncts. *Physiotherapy for Respiratory and Cardiac Problems*.

[44] Menkes H. A., Britt J. (1980). Rationale for physical therapy. *American Review of Respiratory Disease*.

[45] Blaney F., Sawyer T. (1997). Sonographic measurement of diaphragmatic motion after upper abdominal surgery: a comparison of three breathing manoeuvres. *Physiotherapy Theory and Practice*.

[46] Manzano R. M., De Carvalho C. R. F., Saraiva-Romanholo B. M., Vieira J. E. (2008). Chest physiotherapy during immediate postoperative period among patients undergoing upper abdominal surgery: Randomized clinical trial. *Sao Paulo Medical Journal*.

[47] Minschaert M., Vincent J. L., Ros A. M., Kahn R. J. (1982). Influence of incentive spirometry on pulmonary volumes after laparotomy. *Acta Anaesthesiologica Belgica*.

